# Molecular phylogeny and morphology reveal four new species of *Thelephora* (Thelephorales, Basidiomycota) from subtropical China, closely related to *T. ganbajun*

**DOI:** 10.3389/fmicb.2023.1109924

**Published:** 2023-03-14

**Authors:** Shi-Rong Yang, Yu-Lian Wei, Hai-Sheng Yuan

**Affiliations:** ^1^CAS Key Laboratory of Forest Ecology and Management, Institute of Applied Ecology, Chinese Academy of Sciences, Shenyang, China; ^2^University of the Chinese Academy of Sciences, Beijing, China

**Keywords:** ectomycorrhizal fungi, multi-loci phylogeny, taxonomy, Thelephoraceae, new species

## Abstract

The genus of *Thelephora* is a group of cosmopolitan ectomycorrhizal fungi with basidiocarps of morphological diversity that has an extremely scarce species reported from the forest ecosystem in China. In this study, phylogenetic analyses of *Thelephora* species from subtropical China were carried out based on multiple loci including the internal transcribed spacer (ITS) regions, the large subunit of nuclear ribosomal RNA gene (nLSU), and the small subunit of mitochondrial rRNA gene (mtSSU). Maximum likelihood and Bayesian analyses were used to construct the phylogenetic tree. The phylogenetic positions of four new species, *Th. aquila, Th. glaucoflora, Th. nebula*, and *Th. pseudoganbajun*, were revealed based on morphological and molecular evidence. Molecular analyses demonstrated that the four new species were closely related to *Th. ganbajun* and formed a clade with robust support in the phylogenetic tree. Regarding morphology, they share some common morphological characteristics, including flabelliform to imbricate pilei, generative hyphae more or less covered by crystals, and subglobose to irregularly lobed basidiospores (5–8 × 4–7 μm) with tuberculate ornamentation. These new species are described and illustrated and are compared to similar morphological or phylogenetically related species. A key to the new and allied species from China is provided.

## Introduction

*Thelephora* Ehrh. ex Willd. was established by Willdenow with *Thelephora terrestris* Ehrh. as the type species (Willdenow, 1787; Corner, 1968, 1976). The genus along with *Tomentella* Pers. ex Pat., *Polyozellus* Murrill., and *Amaurodon* J. Schröt. were placed in the Thelephoraceae, based on phylogenetic evidence (Larsen, 1968, 1974; Stalpers, 1993; Kõljalg, 1996; Vizzini et al., 2016). In general, this group of the Thelephoraceae has a tremendous variation regarding morphological characteristics, notably distinct color and shape of basidiocarps, as well as diverse size and ornamentation of the basidiospores (Corner, 1968; Larsen, 1968, 1974; Stalpers, 1993; Kõljalg, 1996; Larsson et al., 2004). *Thelephora*, the type genus of the family, also displays considerable diversity in morphology.

The variable morphological and anatomical features of *Thelephora* have been discussed in detail in subsequent studies (Ramírez-López et al., 2013, 2015; Khalid and Hanif, 2017; Das et al., 2018; Li et al., 2020). For instance, the basidiocarps range from stereoid, coral-like, merismatoid, spathulate-rosulate; the hyphal system is generally monomitic; the generative hyphae are usually clamped, simple-septate, and smooth to encrusted; cystidia are often absent; the ornamentation of the basidiospores is usually tuberculate or echinulate (Cunningham, 1957; Corner, 1968; Stalpers, 1993; Ramírez-López et al., 2013, 2015; Khalid and Hanif, 2017; Das et al., 2018; Li et al., 2020). Basidiocarps of some species exhibit phenotypic plasticity, e.g., *Th. versatilis* and *Th. Pseudoversatilis*, can display a sub-resupinate or completely resupinate form over living plants (Ramírez-López et al., 2013, 2015).

Generally, species of *Thelephora* contain a variety of shapes of basidiocarps and basidiospores with tuberculate or echinulate ornamentation, which help differentiate it from other genera in Thelephoraceae. Nevertheless, molecular validations have confirmed that *Thelephora* and *Tomentella* have a very close genetic relationship on the phylogenetic tree; the two genera are usually intermixed in the same evolutionary branch and do not form separate monophyletic groups (Vizzini et al., 2016; Lu et al., 2022). Furthermore, some species of the two genera have similar microscopic characteristics, such as tuberculate or echinulate ornamentation, and the same size and form as basidiospores (Stalpers, 1993; Lee et al., 2010; Yorou et al., 2011; Ramírez-López et al., 2015). The subtle classification boundary of the two genera has been controversial and still remains unresolved (Ramírez-López et al., 2015; Li et al., 2020; Lu et al., 2022).

Members of the genus *Thelephora* play an essential role in ecology, e.g., *Th. terrestris* is a well-known and rather common ectomycorrhizal symbiont in conifer tree nurseries promoting the growth of conifer seedlings (Weir, 1921; Corner, 1968; Marx and Bryan, 1970). Some species possess edible and medicinal values, for instance, *Th. ganbajun* M. Zang is a delicious edible fungus with a high economic value in China. Recent studies have documented that the chemically active ingredients, such as p-biphenyl phenolic compounds, polysaccharides, steroids, and fatty acids, extracted from *Th. ganbajun* have multiple effects such as antioxidant, antitumor, liver protection, and immune system enhancement for humans (Xu et al., 2016; Wang et al., 2017a; Zheng et al., 2020; Lu et al., 2022).

Approximately 52 accepted species of *Thelephora* have been described worldwide (http://www.indexfungorum.org/Names/Names.asp). Several studies have provided an ITS or ITS + LSU phylogenetic overview of the genus (Ramírez-López et al., 2015; Vizzini et al., 2016; Das et al., 2018; Li et al., 2020), based on species from the northern temperate and tropical regions of Asia, Europe, and North America (Ramírez-López et al., 2013, 2015; Khalid and Hanif, 2017; Das et al., 2018; Li et al., 2020). To date, 21 species of *Thelephora* have been recorded from China (Teng, 1934; Li et al., 2020; Liu et al., 2021), and most of them were identified based on themorphological comparison in the last century, and reference to taxonomy and phylogeny of this genus is extremely scarce. Meanwhile, most of the identifications are from molecular sequences without morphological study in the new century. For the star species *Th. ganbajun*, the situation may be even worse, as the name “ganbajun” has been applied to most related sequences in GenBank by some researchers. There are over 600 ITS sequences named “*Thelephora ganbajun*” in the NCBI database, yet the sequence discrepancies range from 1 to 9.5%. Therefore, it is essential to clarify the relationship between *Th. ganbajun* and the species with which it can be easily confused.

Investigations of stipitate aphyllophoroid fungi in China have been carried out in recent decades, and numerous *Thelephora* specimens have been collected. During the study of these specimens, four undescribed species collected from subtropical China were identified by means of morphology and phylogenetic analyses of a three-gene (ITS + nLSU + mtSSU) dataset. In this study, we describe and illustrate these taxa based on morphological and phylogenetic evidence and provide a key to the species of *Thelephora* from China.

## Materials and methods

### Morphological studies

Specimens were deposited at the herbarium of the Institute of Applied Ecology, Chinese Academy of Sciences (IFP). Microscopic procedures follow Cao et al. (2021). Macro-morphological characteristics of basidiocarps were observed under a stereomicroscope (Nikon SMZ 1000: Tokyo, Japan) at 4 × magnification. The observations of microscopic characters were performed on freehand sections of dried basidiocarps, mounted in 3% KOH, and stained in Cotton Blue (test for cyanophilous or acyanophilous reactions) and Melzer's reagent (test for amyloid and dextrinoid reactions). All measurements were studied at magnifications up to 1,000 × using a Nikon Eclipse E600 microscope (Tokyo, Japan) with phase contrast illumination. The following abbreviations are used: IKI = Melzer's reagent; IKI – = neither amyloid nor dextrinoid; KOH = 3% potassium hydroxide; CB = Cotton Blue; CB + = cyanophilous; L = mean spore length (arithmetic average of all spores); W = mean spore width (arithmetic average of all spores); Q = variation in the L/W ratios between the specimens studied; and *n* (a/b) = number of spores. The surface morphology for the basidiospores was observed with a Phenom Prox scanning electron microscope (ESEM, Phenom Prox, FEI, Netherlands) at an accelerating voltage of 20 kV. A thin layer of gold was coated on the samples to avoid charging. Special color terms are from Rayner (1970) and Munsell (2015).

### Molecular study

Genomic DNA was extracted from the dried specimens with a Thermo Scientific Phire Plant Direct PCR kit (Thermo Fisher Scientific, Waltham, MA, United States). The internal transcribed spacer region (ITS) was amplified with primer pairs ITS4 and ITS1-F (White et al., 1990); the large subunit of nuclear ribosomal RNA gene (nLSU) with LR0R and LR5 (Moncalvo et al., 1993); and the mitochondrial small subunit rDNA gene (mtSSU) with MS1 and MS2 (Matheny, 2005). The final PCR volume was 30 μl; each tube contained 0.9 μl of template DNA, 15 μl of 2 × Phire Plant PCR buffer, 1.5 μl of each primer, 0.6 μl pf Phire HS II DNA polymerase, and 10.5 μl of ddH_2_O (double distilled water). The PCR thermal cycling program conditions were as follows: initial denaturation at 95°C for 3 min, followed by 35 cycles of denaturation at 95°C for 40 s; annealing at 54°C for 45 s (ITS), 50°C for 1 min (nLSU), and 43°C for 50 s (mtSSU); extension at 72°C for 1 min; and a final extension at 72°C for 10 min (Yuan et al., 2020; Mu et al., 2021). The PCR products were purified and sequenced at the Beijing Genomics Institute (BGI), China.

### Phylogenetic analyses

The newly generated sequences in this study and related sequences downloaded from GenBank (Table 1) were converted into FASTA format files by ClustalX (Thompson et al., 1997). Then, alignments were performed using MAFFT 7.110 (Katoh et al., 2019) and manually adjusted to allow maximum alignment and minimize gaps; finally, the results of the alignments were saved as the FASTA format files.

**Table 1:** Voucher numbers, geographic origins, and GenBank accession numbers for the specimens included; sequences produced in this study are in bold.

**Species**	**GenBank No./UNITE database accession No**.			**Voucher number**	**Locality**	**References**
	**ITS**	**LSU**	**mtSSU**			
*Odontia fibrosa* (Berk. and M.A. Curtis) Kõljalg	MT981502	MT981502	–	LE F-332368	Russia	NCBI database
*Thelephora anthocephala* (Bull). Fr.	DQ974771	–	–	src614	USA	NCBI database
*Th. anthocephala*	MT773612	MT773612	–	NSK1014540	Russia	NCBI database
*Th*. cf. *anthocephala*	MF926570	MF926570	–	108	Russia	NCBI database
*Th*. cf. *anthocephala*	MF926569	–	–	157	Russia	NCBI database
*Th. albomarginata* Schwein.	UDB01669	–	–	MC01-544	Denmark	UNITE database
*Th. alnii* Kõljalg.	UDB002953	–	–	SH1502191	Estonia	NCBI database
*Th. alnii*	UDB002958	–	–	TH034568	Estonia	NCBI database
*Th. americana* Lloyd	MT196971	–	–	BMJ01	USA	NCBI database
*Th. atramentaria* (Rostr.) Sacc.	UDB000236	–	–	TUF123496	Germany	UNITE database
*Th. atramentaria*	UDB000955	–	–	TUF108866	Estonia	UNITE database
*Th. atrocitrina* Quél.	UDB023357	–	–	SH1502461	Italy	UNITE database
*Th. atrocitrina*	UDB023355	–	–	SH1213711	Italy	UNITE database
***Th. aquila***	**OP793743**	**OP793698**	**OP793724**	**Wei 8831**	**China**	**This sutdy**
***Th. aquila***	**OP793744**	**OP793699**	**OP793725**	**Wei 8833**	**China**	**This sutdy**
*Th. aurantiotincta* Corner	MZ057686	–	–	520625MF420	China	NCBI Database
*Th. aurantiotincta*	AB509809	–	–	346-518	Japan	NCBI Database
*Th. austrosinensis* T.H. Li & T. Li	MF593261	–	–	GDGM 25680	China	Li et al., 2020
*Th. austrosinensis*	MF593265	–	–	GDGM 48867	China	Li et al., 2020
*Th. caryophyllea* (Schaeff.) Pers.	KC152242	–	–	GO-2010-163	Mexico	NCBI database
*Th. caryophyllea*	KR606030	–	–	BSI 13/103	Switzerland	NCBI database
*Th*. cf. *caryophyllea*	MZ890174	–	–	UBC: F29441	Canada	NCBI database
*Th*. cf. *caryophyllea*	MZ890175	–	–	UBC: F29912	Canada	NCBI database
*Th. cuticularis* Berk.	UDB023363	–	–	MCVE23531	Italy	UNITE database
*Th. cuticularis*	UDB023383	–	–	MICH139821	Italy	UNITE database
*Th. dominicana* A. Losi & Vizzini	KX216400	–	–	JBSD126510	Italy	Vizzini et al., 2016
*Th. ganbajun* M. Zang	KY245240	–	–	HKAS 10484	China	Wang et al., 2017b
*Th. ganbajun*	**OP793757**	**OP793791**	**OP793722**	**Yuan 14373**	**China**	**This sutdy**
*Th. ganbajun*	**OP793758**	**OP793792**	**OP793723**	**Yuan 14374**	**China**	**This sutdy**
*Th. ganbajun*	**OP793760**	**OP793685**	**OP793716**	**Yuan 16715**	**China**	**This sutdy**
*Th. ganbajun*	**OP793763**	**OP793686**	**OP793717**	**Yuan 16749**	**China**	**This sutdy**
*Th. ganbajun*	**OP793761**	**OP793790**	**OP793718**	**Yuan 16756**	**China**	**This sutdy**
*Th. ganbajun*	**OP793764**	**OP793689**	**OP793719**	**Yuan 16765**	**China**	**This sutdy**
*Th. ganbajun*	**OP793759**	**OP793688**	**OP793720**	**Yuan 16769**	**China**	**This sutdy**
*Th. ganbajun*	**OP793762**	**OP793687**	**OP793721**	**Yuan 16817**	**China**	**This sutdy**
***Th. glaucoflora***	**OP793751**	**O793696**	**OP793730**	**Dai 13623A**	**China**	**This sutdy**
***Th. glaucoflora***	**OP793752**	**OP793697**	**OP793731**	**Dai 13627A**	**China**	**This sutdy**
***Th. glaucoflora***	**OP793753**	**–**	**OP793734**	**Dai 15217**	**China**	**This sutdy**
***Th. glaucoflora***	**OP793754**	**–**	**–**	**Dai 16612**	**China**	**This sutdy**
***Th. glaucoflora***	**OP793755**	**OP799736**	**OP793735**	**Dai 19753**	**China**	**This sutdy**
***Th. glaucoflora***	**OP793756**	**OP793695**	**OP793733**	**He 3868**	**China**	**This sutdy**
***Th. glaucoflora***	**OP793750**	**–**	**OP793732**	**He 4441**	**China**	**This sutdy**
***Th. glaucoflora*** as “*Th. ganbajun*”	EU696818	**–**	**–**	Gb046	China	NCBI database
***Th. glaucoflora*** as “*Th. ganbajun*”	EU696831	**–**	**–**	Gb082	China	NCBI database
***Th. glaucoflora*** as “*Th. ganbajun*”	EU696871	**–**	**–**	Gb149	China	NCBI database
***Th. glaucoflora*** as “*Th. ganbajun*”	EU696881	**–**	**–**	Gb160	China	NCBI database
*Th. grandinioides* C.L. Zhao & X.F. Liu	MZ400673	MZ400675	–	CLZhao 3406	China	Liu et al., 2021
*Th. grandinioides*	MZ400674	MZ400676	–	CLZhao 3408	China	Liu et al., 2021
*Th. iqbalii* Khalid & Hanif	JX241471	JX241471	–	MH810	Pakistan	Khalid and Hanif, 2017
*Th. mollissima* Pers.	UDB023356	–	–	–	Italy	UNITE database
***Th. nebula***	**OP793745**	**–**	**–**	**Yuan 11515**	**China**	**This sutdy**
***Th. nebula***	**OP793746**	**OP793694**	**OP793729**	**Yuan 11516**	**China**	**This sutdy**
***Th. nebula***	**OP793747**	**–**	**OP793728**	**Yuan 11518**	**China**	**This sutdy**
***Th. nebula***	**OP793748**	**OP793693**	**OP793727**	**He 4452**	**China**	**This sutdy**
***Th. nebula***	**OP793749**	**–**	**OP793726**	**He 4456**	**China**	**This sutdy**
*Th. palmata* (Scop.) Fr.	UDB018570	–	–	AT2005162	Sweden	NCBI database
*Th. palmata*	MH310778	–	–	TU115271	Sweden	NCBI database
*Th. penicillata* (Pers.) Fr.	OL469898	OL469898	–	X618	Prague	Borovička et al., 2022
*Th. penicillata*	OL469899	OL469899	–	X619	Prague	Borovička et al., 2022
*Th*. aff. *penicillata*	KP814285	KP814285	–	UC2022955	USA	NCBI database
***Th. pseudoganbajun*** as “*Th*. cf. *ganbajun*”	KY245247	–	–	XY18-3	China	Wang et al., 2017b
***Th. pseudoganbajun*** as “*Th*. cf. *ganbajun*”	KY245255	–	–	YL3-10	China	Wang et al., 2017b
***Th. pseudoganbajun*** as “*Th. ganbajun*”	EU696860	–	–	Gb124	China	NCBI database
***Th. pseudoganbajun*** as “*Th. ganbajun*”	EU696931	–	–	Gb263	China	NCBI database
***Th. pseudoganbajun***	**OP793770**	**OP793701**	**OP793711**	**Yuan 16733**	**China**	**This sutdy**
***Th. pseudoganbajun***	**OP793765**	**OP793705**	**OP793715**	**Yuan 16771**	**China**	**This sutdy**
***Th. pseudoganbajun***	**OP793766**	**OP793703**	**OP793713**	**Yuan 16780**	**China**	**This sutdy**
***Th. pseudoganbajun***	**OP793768**	**OP793700**	**OP793710**	**Yuan 16794**	**China**	**This sutdy**
***Th. pseudoganbajun***	**OP793767**	**OP793702**	**OP793712**	**Yuan 16835**	**China**	**This sutdy**
*Th. pseudoterrestris* corner	UDB000209	–	–	TAAM159625	Thailand	UNITE database
*Th. pseudoterrestris*	AF272907	–	–	TAA159625	Sweden	NCBI database
*Th. pseudoversatilis* Ram.-Lóp. & Villegas	JX075890	–	–	FCME 26232	Mexico	NCBI database
*Th. pseudoversatilis*	KJ462486	–	–	FCME 26152	Mexico	NCBI database
*Th. regularis* Schwein.	MG953966	–	–	UBC F33227	Canada	NCBI database
*Th. regularis*	U83485	–	–	JMT17371	USA	NCBI database
*Th*. aff. *regularis*	KC152240	–	–	GO-2010-125	Mexico	NCBI database
*Th*. aff. *regularis*	KC152241	–	–	GO-2010-134	Mexico	NCBI database
*Th. scissilis* Burt	OK376730	–	–	MUOB:324045	USA	NCBI database
*Th. sikkimensis* K. Das, Hembrom & Kuhar	MF684017	MF684017	–	KD 16-003	India	Das et al., 2018
*Th. sikkimensis*	MF684018	–	–	KD 16-042	India	Das et al., 2018
*Th. sublilacina* Ellis & Everh.	EF493288	–	–	UP161	Sweden	NCBI database
*Th. terrestris* Ehrh.	DQ822828	DQ822828	–	KGP22	USA	NCBI database
*Th. terrestris*	FJ532478	–	–	Hilszczanska D. 1-IBL	Poland	NCBI database
*Th*. cf. *terrestris*	KT800293	–	–	7	USA	NCBI database
*Th*. cf. *terrestris*	KT800086	–	–	177	USA	NCBI database
*Th. vialis* Schwein.	MN121022	MN121022	–	TENN-F-072094	USA	NCBI database
*Th. vialis*	MN121029	MN121029	–	TENN-F-072281H2	USA	NCBI database
*Th. versatilis* Ram.-Lóp. & Villegas	NR154492	–	–	UNAM: FCME26141	Mexico	NCBI database
*Th. versatilis*	KJ462504	KJ462504	–	UNAM: FCME26141	Mexico	NCBI database
*Th. wuliangshanensis* C.L. Zhao & X.F. Liu	MZ400671	MZ400677	–	CLZhao 4107	China	Liu et al., 2021
*Th. wuliangshanensis*	MZ400672	MZ400678	–	CLZhao 21020	China	Liu et al., 2021
*Tomentella atrobadia* H.S. Yuan & Y.C. Dai	KY686248	MK446335	–	Yuan 11099	China	Yuan et al., 2020
*T. atrobadia*	KY686249	MK446336	–	Yuan 11114	China	Yuan et al., 2020
*T. citrinocystidiata* H.S. Yuan & Y.C. Dai	KY686246	MK446348	–	Yuan 10680	China	Yuan et al., 2020
*T. storea* H.S. Yuan & Y.C. Dai	KY696245	MK446416	–	Yuan 10749	China	Yuan et al., 2020

The combined ITS + nLSU + mtSSU dataset phylogenetic analyses were conducted using maximum likelihood (ML) and Bayesian inference (BI) analysis. All characters were equally weighted, and all gaps were treated as missing data. ModelFinder (Kalyaanamoorthy et al., 2017) on Phylosuite (Zhang et al., 2020) was used to select the best-fit partition model (Edge-linked) using the AICc criterion for combined ITS + nLSU + mtSSU dataset. Best-fit models according to AICc were as follows: K3Pu + F + I + G4 (ITS), TIM3 + F + R2 (nLSU), TIM + F + I (mtSSU) for ML; SYM + I + G4 (ITS), GTR + F + I + G4 (nLSU), and GTR + F + I (mtSSU) for BI. Maximum likelihood phylogenies were inferred using IQ-TREE (Nguyen et al., 2015) under the edge-linked partition model for 1,000 standard bootstraps, as well as the Shimodaira–Hasegawa-like approximate likelihood-ratio test (Guindon et al., 2010). Bayesian Inference phylogenies were inferred using MrBayes 3.2.6 (Ronquist et al., 2012) implementing the Markov Chain Monte Carlo technique, with two parallel runs and eight million replicates. Four simultaneous chains were run beginning from random trees, and sampling one tree for every 100 generations until the average standard deviation of split frequencies was below 0.01. The burn-in was set to discard 25% of the trees. Identity/similarity between two sequences was calculated using the “pairwise alignment, calculation of the similarity/identity” option of BioEdit v. 7.0.5 (Hall, 2005).

## Results

### Phylogenetic analyses

The combined 143 ITS + nLSU + mtSSU sequences representing 41 taxa were used to build phylogenetic trees; 73 sequences of *Thelephora* were newly generated, 70 sequences were downloaded from GenBank (Table 1), including eight sequences of *Tomentella*. *Odontia ferruginea* was used as the outgroup (Ramírez-López et al., 2015; Vizzini et al., 2016; Khalid and Hanif, 2017; Das et al., 2018; Yuan et al., 2018; Li et al., 2020; Liu et al., 2021; Borovička et al., 2022).

**Figure 1:**
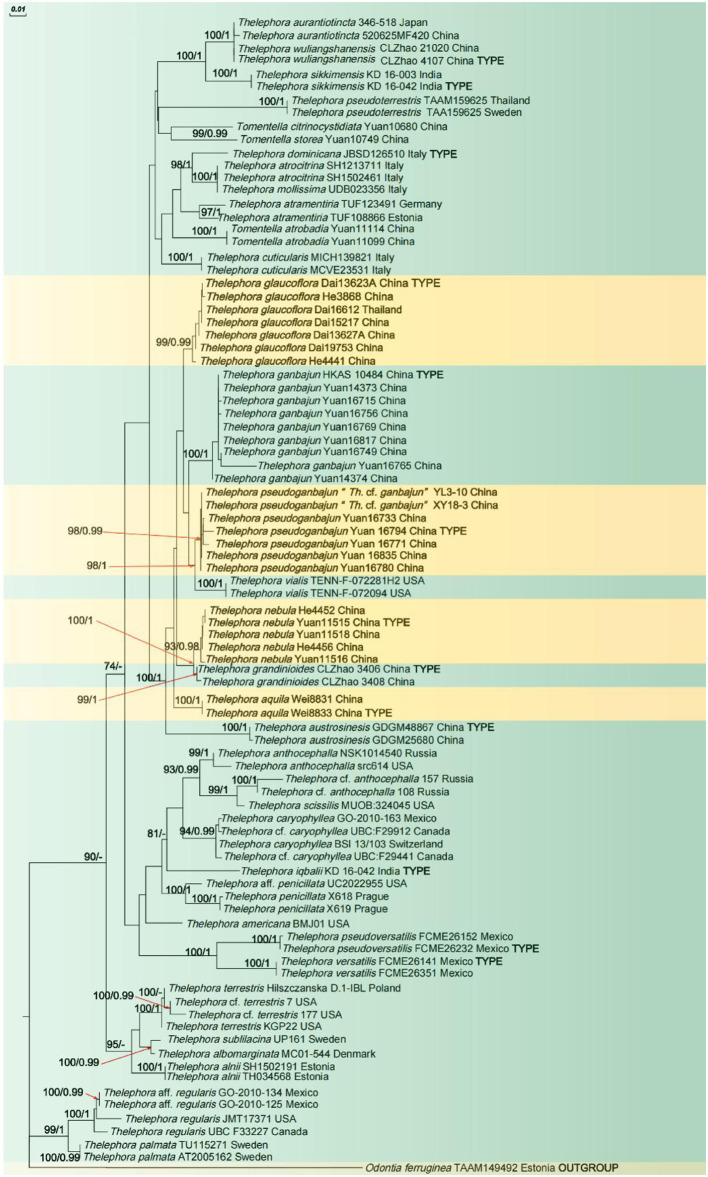
ML tree illustrating the phylogeny of the new species and related taxa based on ITS + nLSU + mtSSU nuclear rDNA sequences dataset. Branches are labeled with maximum likelihood bootstrap higher than 50% and Bayesian posterior probabilities higher than 0.95.

In the phylogenetic tree (Figure 1), 19 sampled specimens representing four new species formed four isolated clades with strong support (100% ML/1 BI for *Th. aquila*, 99% ML/0.99 BI for *Th. glaucoflora*, 93% ML/0.98 BI for *Th. nebula*, and 98% ML/0.99 BI for *Th. pseudoganbajun*) and clustered in the clade with other four species including *Th. austrosinensis, Th. ganbajun, Th. grandinioides*, and *Th. vialis* with strong support (100% ML/1 BI). Eight samples of *Th. ganbajun* and the type samples formed a fully supported lineage (100% ML/1 BI) that differs from the other samples. The phylogenetic tree also reveals that three taxa of *Tomentella* and 37 taxa of *Thelephora* are intermixed in the phylogenetic tree.

## Taxonomy

***Thelephora aquila*** S.R. Yang, Y.L. Wei & H.S. Yuan, sp. nov.

**Figure 2:**
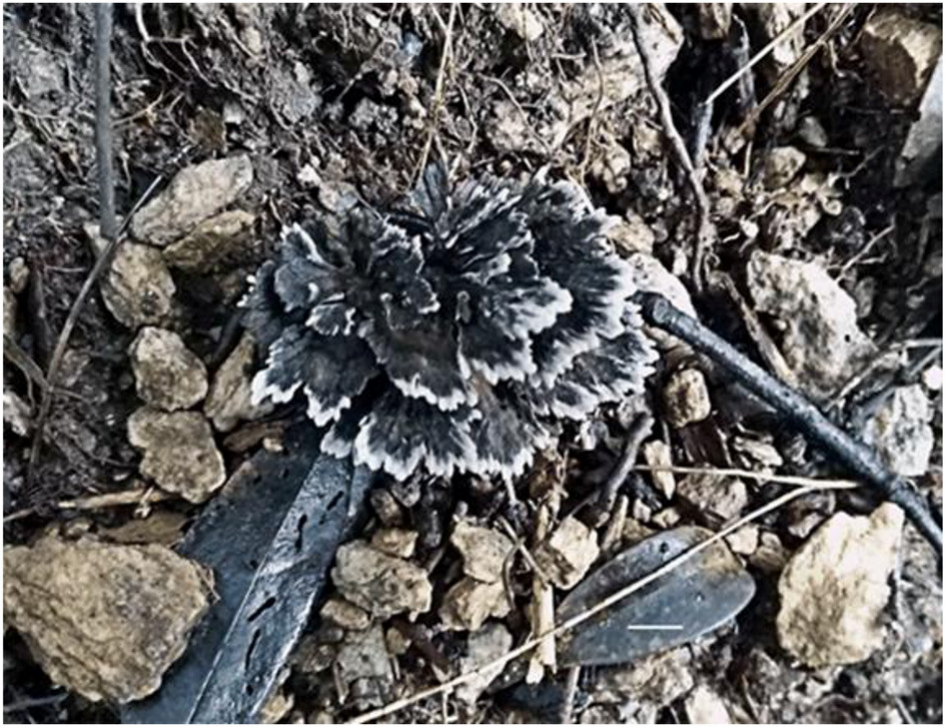
Basidiocarp of *Thelephora aquila* (IFP 19531).

**Figure 3:**
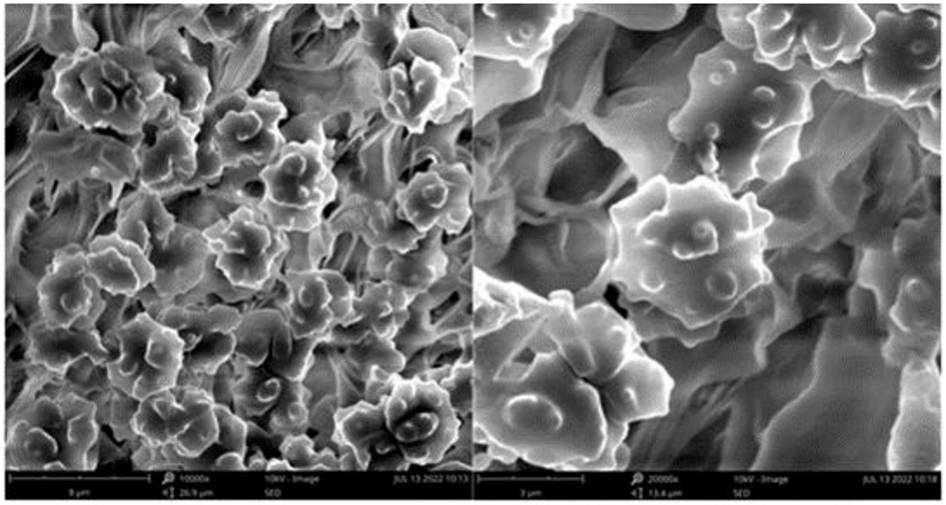
SEM of basidiospores of *Thelephora aquila* (IFP 19531).

**Figure 4:**
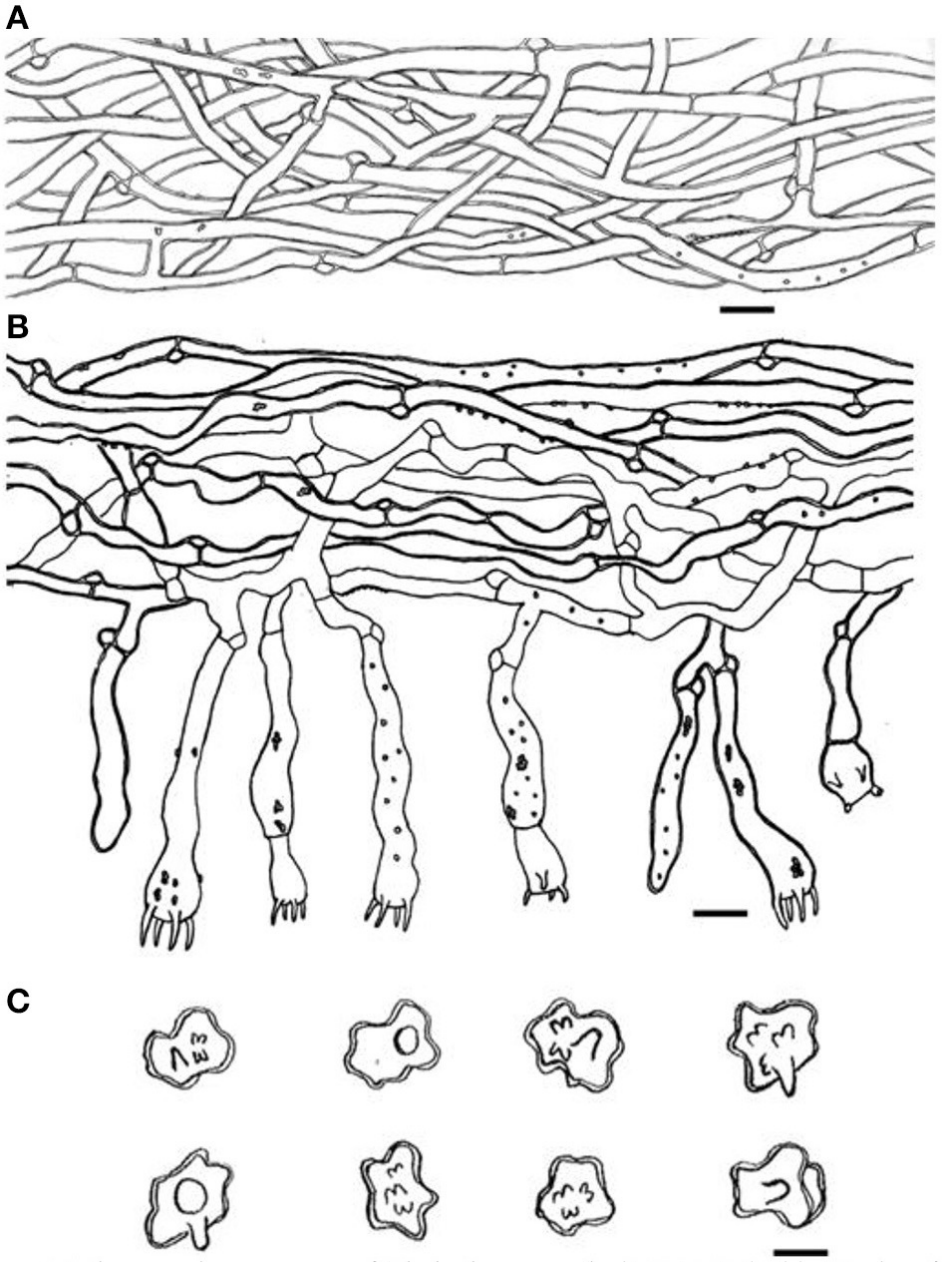
Microscopic structures of *Thelephora aquila* (IFP 19531). **(A)** Hyphae from pileal context; **(B)** section of hymenium and subhymenium; **(C)** basidiospores. Scale bars: **(A, B)** = 10 μm; **(C)** = 5 μm.

MycoBank MB846422 (Figures 2–4).

Diagnosis. Basidiocarps upright, flabelliform to applanate-lobate; abhymenial surface black, margin lobed to wavy; stipe central. Generative hyphae are commonly clamped and rarely simple-septate. Basidiospores are subglobose to irregularly lobed and tuberculate.

Type. CHINA. Zhejiang Province, Kaihua County, Gutianshan National Nature Reserve, 29°16′N, 118°17′E, elev. 961 m, growing in broad-leaved forests, 26.VII.2018, Wei 8833 (holotype IFP 19531).

Etymology. *Aquila* (Lat.), refers to dark-colored, almost black basidiocarps.

Basidiocarp: Upright, solitary to concrescent, small to medium-sized, up to 5 cm high and 4 cm wide, coriaceous when fresh, hard and light in weight when dried; taste mild, almost no odor when dry. Pileus flabelliform to applanate-lobate, usually with multiple pilei arising from a central common stipe or base, margin thin (0.1 mm), irregularly lobed to wavy. Abhymenial surface somewhat radially rugulose or wrinkled, zonate, black (GLEY 1 2.5/N) near the center then gradually turning pale toward the outside and becoming white (30A1) at the margin when fresh; hymenial surface concolorous or deeper and white (30A1) at the margin. Stipe central, up to 2 cm long, brown (6E8), glabrous, clavillose to flatted or broadened.

Hyphal structure: hyphal system monomitic; generative hyphae commonly clamped and rarely simple-septate, CB+ in thick-walled hyphae, IKI–; tissues turned brown-black in 3% KOH.

Context: Generative hyphae hyaline, thick-walled (< 1 μm), moderately branched, sometimes flexuous and collapsed, subparallel to loosely interwoven, 3–6 μm in diam.

Subhymenium: Generative hyphae hyaline, slightly thick-walled (< 1 μm), frequently branched often near the clamp connections, distinctly inflated, occasionally covered by dense crystals, loosely interwoven, up to 4–7 μm diam.

Cystidia and cystidioles: Absent.

Basidia: utriform to subcylindrical, thin- to slightly thick-walled (< 1 μm), 40–55 × 6–10 μm, clamped at the base, multi-guttulate content sometimes very dense, occasionally covered by dense crystals, with four sterigmata, sterigmata 2–6 μm long and 1–2 μm diam at the base.

Basidiospores: slightly thick-walled (< 1 μm), (5–)5.1–7.1(−7.3) × (4–)4.1–6.1(−6.5) μm (ornamentation excluded), L = 6.3 μm, W = 5.7 μm, Q = 1.11–1.26 (*n* = 60/2), subglobose to irregularly lobed, tuberculate, bluish green in 3% KOH and in distilled water, CB+, IKI–, tuberculi usually isolated, sometimes in groups of two or more.

Additional specimen (paratype) examined: CHINA. Zhejiang Province, Kaihua County, Gutianshan National Nature Reserve, 29°16′N, 118°17′E, elev. 961 m, growing in broad-leaved forests, 26.VII.2018, Wei 8831 (IFP 19532).

Notes: *Thelephora aquila*, together with *Th. austrosinensis, Th. ganbajun, Th. glaucoflora, Th. grandinioides, Th. nebula, Th. Pseudoganbajun*, and *Th. vialis* clustered in a clade with full support based on the molecular evidence (Figure 1). Morphologically, a special characteristic of *Th. aquila* is the black abhymenial surface when mature, which makes it distinct from other species in the genus. Furthermore, *Th. aquila* resembles *Th. austrosinensis* in having single to concrescent basidiocarps, flabelliform to lobate pilei, absence of cystidia, and tuberculate basidiospores. However, *Th. austrosinensis* differs from *Th. aquila* by a grayish black to grayish yellow abhymenial surface and a violet pale gray-yellow hymenial surface (Li et al., 2020). *Th. aquila* and *Th. grandinioides* share some common features, including the upright basidiocarp, flabelliform to applanate-lobate pilei, and tuberculate basidiospores. Nevertheless, *Th. grandinioides* can be differentiated by a fawn to isabelline abhymenial surface, a grandinoid, olivaceous buff to clay-buff hymenial surface, as well as the presence of cystidia (Liu et al., 2021).

***Thelephora glaucoflora*** S.R. Yang & H.S. Yuan, sp. Nov.

**Figure 5:**
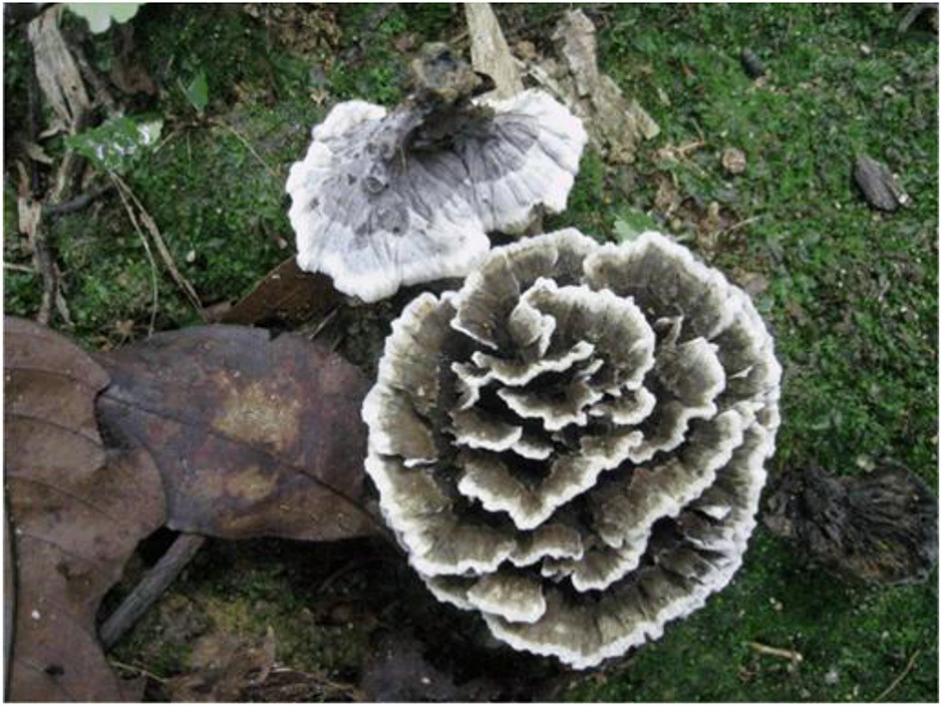
Basidiocarps of *Thelephora glaucoflora* (IFP 19533).

**Figure 6:**
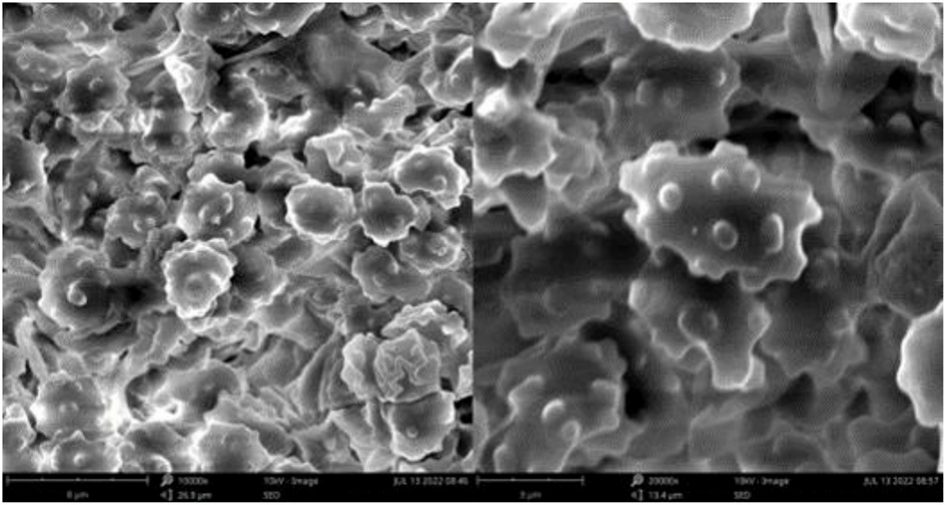
SEM of basidiospores of *Thelephora glaucoflora* (IFP 19533).

**Figure 7:**
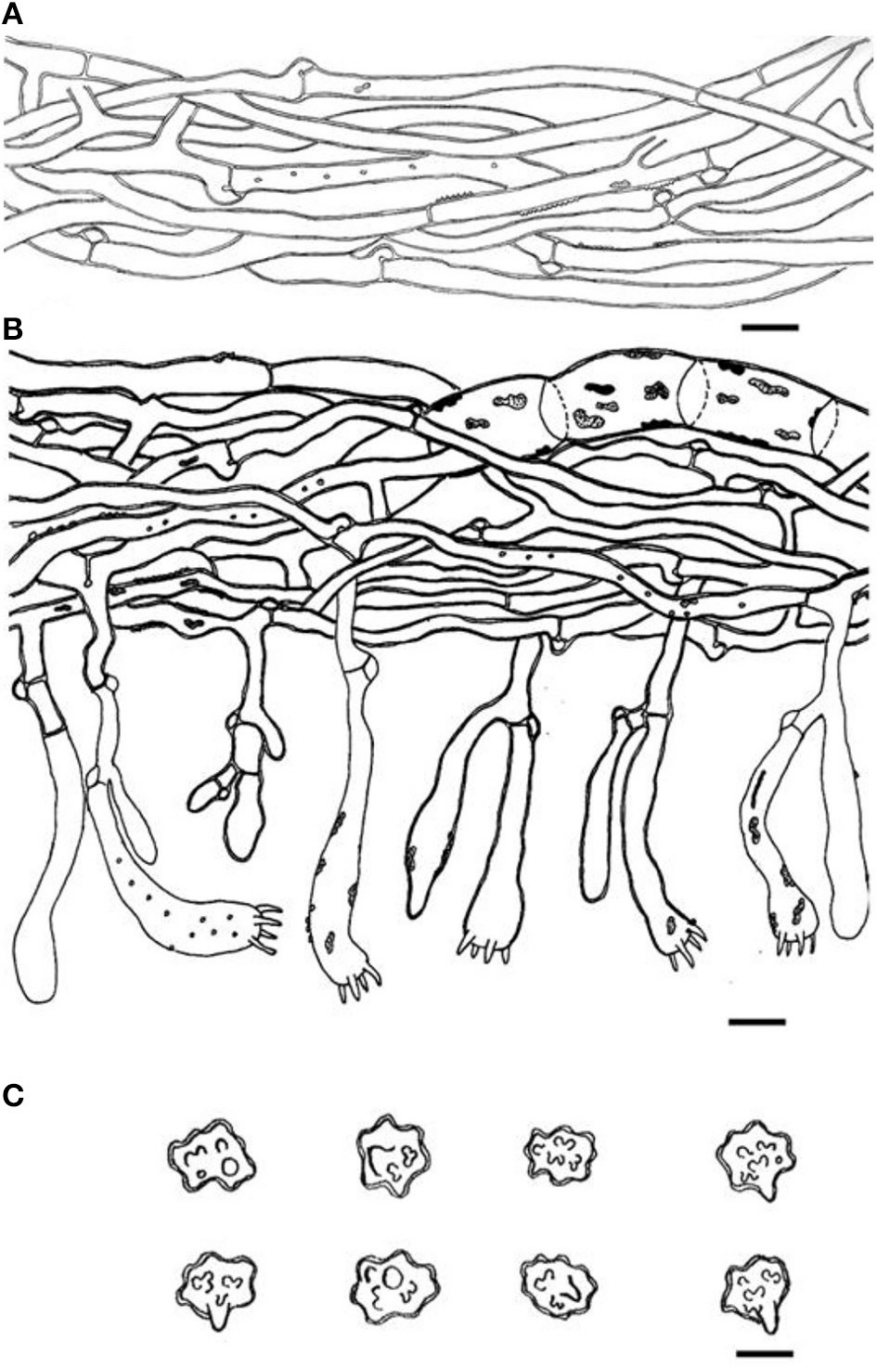
Microscopic structures of *Thelephora glaucoflora* (IFP 19533). **(A)** Hyphae from context; **(B)** section of hymenium and subhymenium; **(C)** basidiospores. Scale bars: **(A, B)** = 10 μm; **(C)** = 5 μm.

MycoBank MB846423 (Figures 5–7).

Diagnosis. Basidiocarps upright, pileus flabelliform, imbricate; abhymenial surface gray to greenish gray, somewhat radially rugulose or wrinkled, zonate, sulcate near the base; stipe short and central. Generative hyphae are commonly clamped and rarely simple-septate, occasionally covered by dense crystals. Basidiospores are subglobose to irregularly lobed, tuberculate.

Type. CHINA. Hainan Province, Qiongzhong County, Limushan National Forest Park, 19°27′N, 109°79′E, elev. 1,412 m, growing on the ground in *Castanopsis* spp. and bamboo forests, 15.VI.2014, Dai 13623A (holotype IFP 19533).

Etymology. *Glaucoflora* refers to the floral basidiocarps with glaucous abhymenial surface.

Basidiocarp: Upright, usually solitary, small to medium-sized, up to 4 cm high and 3 cm wide, coriaceous when fresh, tastes mild, almost no odor when dried. Pileus flabelliform, imbricate, usually with multiple pilei arising from a central stipe or base, basidiocarp like a rose flower, margin thin (0.1 mm thick), imperceptibly wavy. Abhymenial surface somewhat radially rugulose or wrinkled, zonate, sulcate near the base, gray to greenish gray (29E1–29E2) near the center then gradually becoming gray to white (29A1–29B1) at the margin when fresh; hymenial surface radial rugulose, zonate, violet-gray (16B2–16E2) at the base then gray (16D1–16E1), gradually toward margin becoming white (16A1) when fresh. Stipe short and central, up to 0.5 cm long, brown (6E8) to dark brown (6F4–6F8), glabrous, cylindrical to flatted or broadened.

Hyphal structure: Hyphal system monomitic; generative hyphae commonly clamped and rarely simple-septate, CB+ in thick-walled hyphae, IKI–; tissues turned black in 3% KOH.

Context: Generative hyphae hyaline, thin- to slightly thick-walled (< 1 μm), moderately branched, sometimes flexuous and collapsed, subparallel to loosely interwoven, 3–6 μm diam.

Subhymenium: Generative hyphae hyaline, slightly thick-walled (< 1 μm), frequently branched often near the clamp connections, slightly inflated, occasionally covered by dense crystals, loosely interwoven, up to 4–6 μm diam.

Cystidia and cystidioles: Absent.

Basidia: Clavate, thin- to slightly thick-walled (< 1 μm), 40–60 × 6–10 μm, clamped at the base, multi-guttulate content sometimes present, occasionally covered by dense crystals, with four sterigmata, sterigmata 2–6 μm long and 1–2 μm in diam at the base.

Basidiospores: Slightly thick-walled (< 1 μm), (5.1–)5.5–7(−7.1) × (4–)4.5–6(−6.3) μm (ornamentation excluded), L = 6.14 μm, W = 5.08 μm, Q = 1.20–1.21 (*n* = 60/2), subglobose to irregularly lobed, tuberculate, bluish green in 3% KOH and in distilled water, CB+, IKI–, tuberculi usually isolated, sometimes in groups of two or more.

Additional specimens (paratypes) examined: CHINA. Hainan Province, Qiongzhong County, Limushan National Forest Park, 19°27′N, 109°79′E, elev. 1,412 m, growing on the ground in *Castanopsis* spp. and bamboo forests, 15.VI.2014, Dai 13627A (IFP 19534); growing on the ground, 30.V.2015, Dai 15217 (IFP 19535); 8.VI.2016, He 3868 (IFP 19536); Jiangxi Province, Anyuan County, Sanbaishan Scenic Area, 24°59′N, 115°25′E, elev. 1,164.5 m, growing on the ground, 5.VIII.2016, He 4441 (IFP 19537); Yunnan Province, Jinping County, Fenshuiling Nature Reserve, 22°54′N, 103°13′E, elev. 990–3,074.3 m, growing on the ground, 25.VI.2019, Dai 19753 (IFP 19538). THAILAND. Chiang Rai, Mae Fah Luang University, growing on the root of bamboo, 21.VII.2016, Dai 16612 (IFP 19539).

Notes: *Thelephora glaucoflora* has an adjacent phylogenetic relationship with *Th. ganbajun* (Figure 1). In morphology, *Th. glaucoflora* resembles *Th. ganbajun* in having single to concrescent basidiocarps, flabelliform to lobate pilei, bluish green, and tuberculate basidiospores. However, *Th. glaucoflora* differs from the latter by a glaucous to greenish gray abhymenial surface, a violet-gray hymenial surface, a brown to dark brown and non-branched stipe as well as the absence of cystidia (Zang, 1987). *Th. glaucoflora* and *Th. cuticularis* share similar morphological characteristics, including multiple flabelliform to imbricate pilei and the absence of cystidia. Nevertheless, *Th. cuticularis* can be separated from *Th. glaucoflora* by a jet black abhymenial surface, a dark to purplish brown hymenial surface, and bigger, yellowish brown to pale brown basidiospores (8–12 × 6–10 μm in *Th. cuticularis* vs. 5.1–7.1 × 4–6.3 μm in *Th. glaucoflora*) with echinulate ornamentation (Baici et al., 1995). In morphology, *Th glaucoflora* and *Th. dominicana* exhibit some common features, including the absence of cystidia and central stipe. However, *Th. dominicana* differs from *Th. glaucoflora* by a black to grayish abhymenial surface, a grayish to vinaceous gray hymenial surface, a black cylindrical stipe, infundibuliform pileus, and dark brown basidiospores with echinulate ornamentation (Vizzini et al., 2016).

***Thelephora nebula*** S.R. Yang & H.S. Yuan, sp. nov.

**Figure 8:**
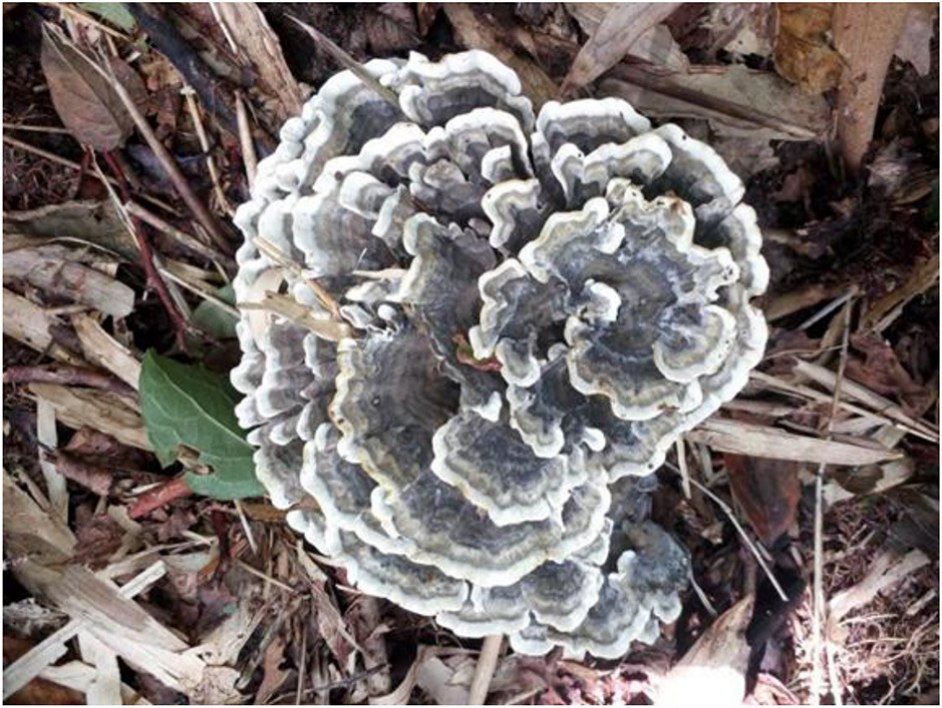
Basidiocarp of *Thelephora nebula* (IFP 19540).

**Figure 9:**
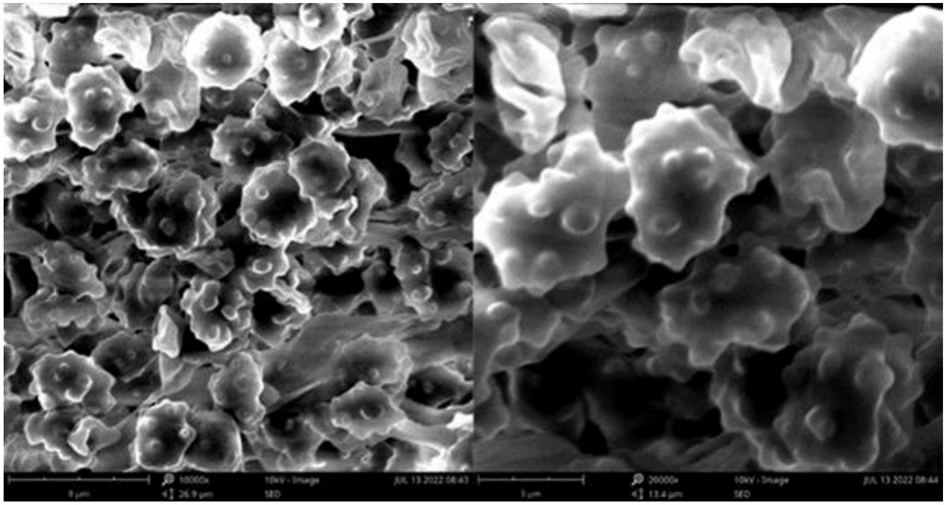
SEM of basidiospores of *Thelephora nebula* (IFP 19540).

**Figure 10:**
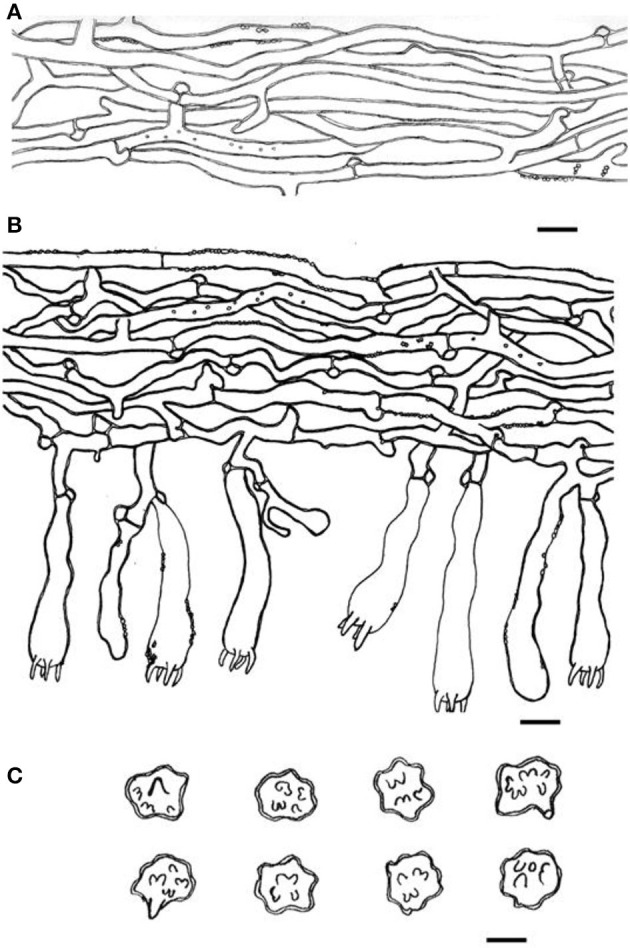
Microscopic structures of *Thelephora nebula* (IFP 19540). **(A)** Hyphae from pileal context; **(B)** section of hymenium and subhymenium; **(C)** basidiospores. Scale bars: **(A, B)** = 10 μm; **(C)** = 5 μm.

MycoBank MB846424 (Figures 8–10).

Diagnosis. Basidiocarps upright, pileus flabelliform to applanate-lobate, imbricate; abhymenial surface grayish brown to yellowish brown, margin lobed to wavy; stipe short and central. Generative hyphae are commonly clamped and rarely simple-septate, occasionally covered by dense crystals. Basidiospores globose to subglobose, irregularly lobed, tuberculate.

Type. CHINA. Fujian Province, Nanping City, Wuyishan Nature Reserve, 24°30′N−28°20′N, 115°33′E−118°50′E, elev. 2,158 m, growing in broad-leaved forests, Yuan 11515 (holotype IFP 19540).

Etymology. *Nebula* (Lat.), refers to the basidiocarp resembling a wavy cloud.

Basidiocarp: Upright, solitary to concrescent, medium to large-sized, up to 8 cm high and 10 cm wide, coriaceous when fresh, tastes mild, almost no odor when dried. Pileus flabelliform to applanate-lobate, imbricate, usually with multiple pilei arising from a central stipe or multiple pilei overlapping and fused to form a conical cluster, margin slightly thin (0.1–1 mm), irregularly lobed to obtuse. The abhymenial surface somewhat wrinkled, visibly zonate, sulcate, brown (6E8) to grayish brown (5D3–5F3) near the center then yellowish brown (5E8) gradually toward the margin, brownish yellow (5C3–5C7) to white (5A1) at the margin; hymenial surface zonate, grayish brown (5D3–5F3) to brown (6E8) and toward margin becoming brownish yellow (5C3–5C7) to white (5A1) at the margin. Stipe short and central, up to 2 cm long, brown (6E8), glabrous, clavillose to flatted or broadened.

Hyphal structure: Hyphal system monomitic; generative hyphae commonly clamped and rarely simple-septate, CB+ in thick-walled hyphae, IKI–; tissues turned black in 3% KOH.

Context: Generative hyphae hyaline, slightly thick-walled (< 1 μm), moderately branched, sometimes flexuous and collapsed, subparallel to loosely interwoven, 3–5 μm in diam.

Subhymenium: Generative hyphae hyaline to semi-hyaline, slightly thick-walled (< 1 μm), frequently branched often near the clamp connections, slightly inflated, occasionally covered by dense crystals, loosely interwoven, up to 4–6 μm in diam.

Cystidia and cystidioles: Absent.

Basidia: Utriform to subcylindrical, thin- to slightly thick-walled (< 1 μm), 45–65 × 8–11 μm, clamped at the base, multi-guttulate content sometimes very dense, occasionally covered by dense crystals, with four sterigmata, sterigmata 4–7 μm long and 1–2 μm in diam at the base.

Basidiospores: Slightly thick-walled (< 1 μm), (6–)6.1–7.9(−8) × (4.9–)5–6.5(−7) μm (ornamentation excluded), L = 7.06 μm, W = 5.81 μm, Q = 1.22–1.24 (*n* = 60/2), subglobose to irregularly lobed, tuberculate, yellowish brown to pale green in 3% KOH and in distilled water, CB+, IKI–, tuberculi usually isolated, sometimes in groups of two or more.

Additional specimens (paratypes) examined: **CHINA**. Fujian Province, Nanping City, Wuyishan Nature Reserve, 27°45′N, 118°03′E, elev. 2,158 m, growing in broad-leaved forests, Yuan 11516 & 11518 (IFP 19541 & 19542); 17.VIII.2016, He 4452 & 4456 (IFP 19543 & 19544).

Notes: *Thelephora nebula* formed a distinct lineage that was separated from the clade of *Th. grandinioides* in the phylogenetic tree (Figure 1). Morphologically, *Th. nebula* shares similar features with *Th. grandinioides* by multiple flabelliform to imbricate pilei and tuberculate basidiospores. Nevertheless, *Th. grandinioides* can be differentiated by a fawn to isabelline abhymenial surface, a grandinoid, olivaceous buff to the clay-buff hymenial surface when fresh, as well as the presence of cystidia (Liu et al., 2021). *Th. nebula* and *Th. vialis* share common features including flabelliform to effuse-reflexed pilei, absence of cystidia, and frequently branched subhymenial hyphae. Nevertheless, the diagnostic feature to distinguish the new species is the brown to grayish brown abhymenial surface, grayish brown to brownish yellow hymenial surface, as well as brown and central stipe (Corner, 1968). *Th. nebula* and *Th. palmata* also share some similar features, including multiple flabelliform to the applanate-lobate pilei and the absence of cystidia. However, *Th. palmata* possesses a chocolate brown to blackish brown abhymenial surface, fuscous purple, and bigger echinulate basidiospores (8–12 × 7–9 μm in *Th. palmata* vs. 6.1–7.9 × 5–6.5 μm in *Th. nebula*) as well as bigger basidia (70–100 × 9–12 μm in *Th. palmata* vs. 45–65 × 8–11 μm in *Th. nebula* Corner, 1968).

***Thelephora pseudoganbajun*** S.R. Yang & H.S. Yuan, sp. nov.

**Figure 11:**
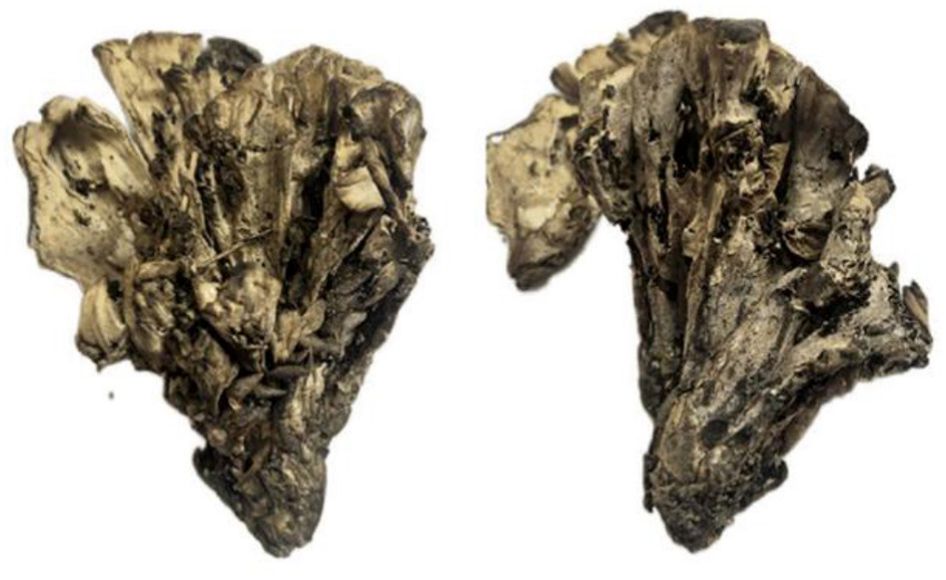
Basidiocarps of *Thelephora pseudoganbajun* (IFP 19545).

**Figure 12:**
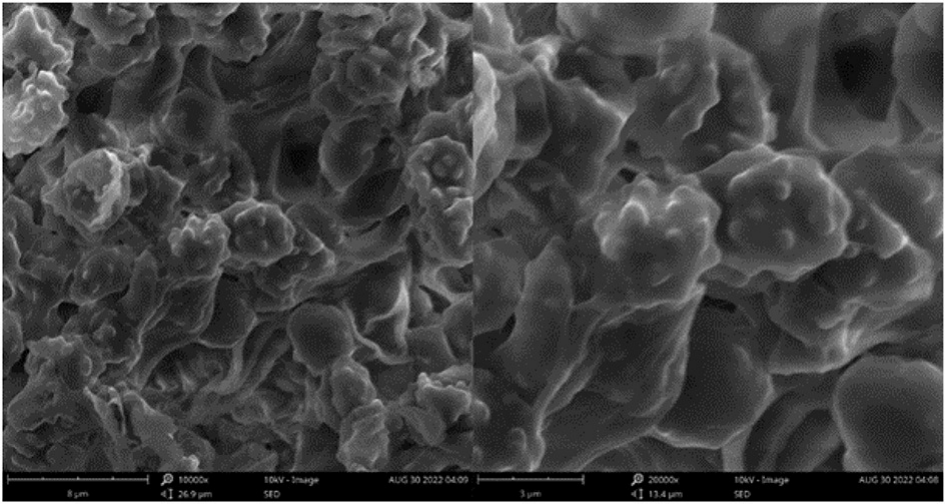
SEM of basidiospores of *Thelephora pseudoganbajun* (IFP 19545).

**Figure 13:**
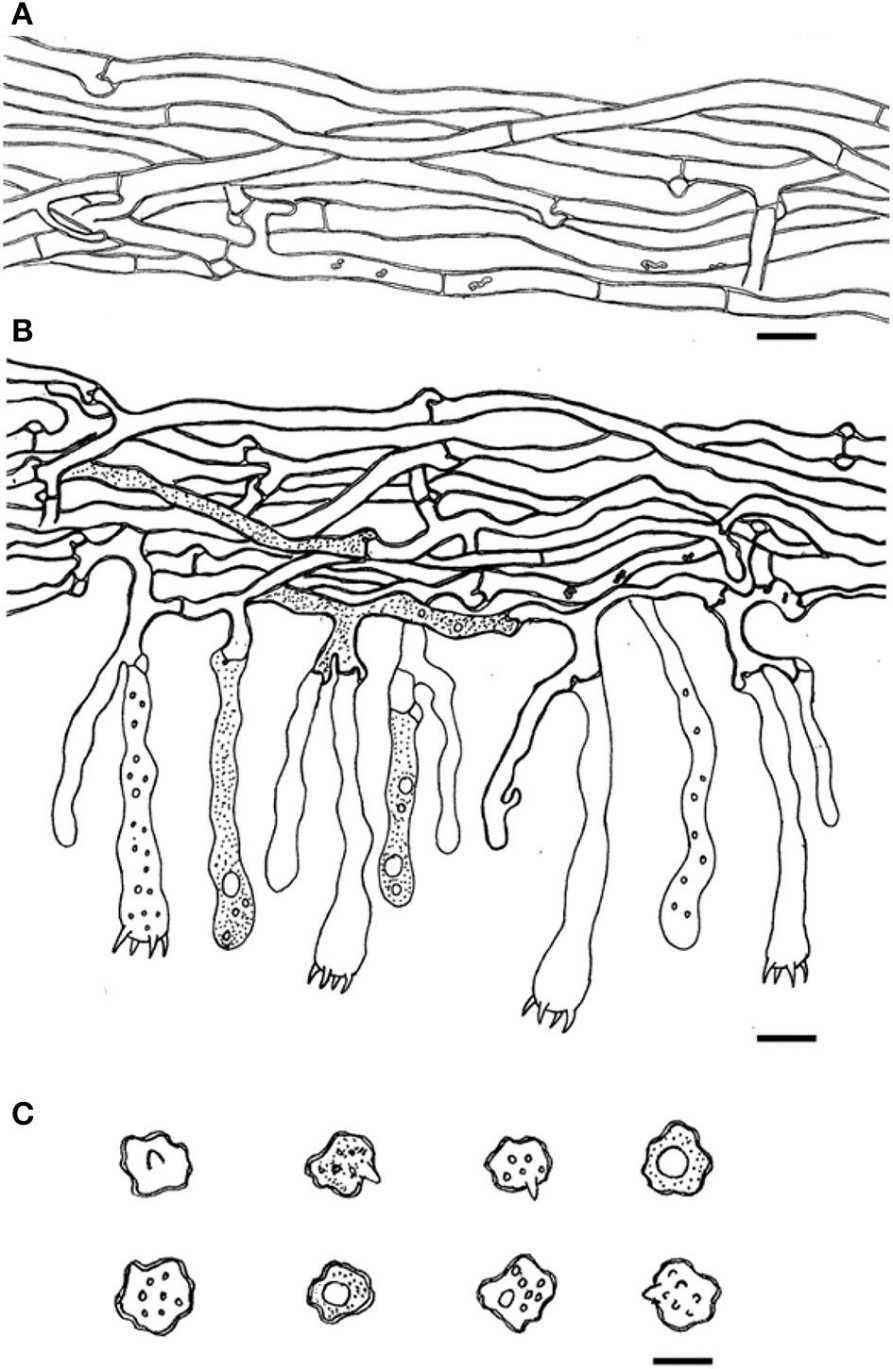
Microscopic structures of *Thelephora pseudoganbajun* (IFP 19545). **(A)** Hyphae from pileal context; **(B)** section of hymenium and subhymenium; **(C)** basidiospores. Scale bars: **(A, B)** = 10 μm; **(C)** = 5 μm.

MycoBank MB846425 (Figures 11–13).

Diagnosis. Basidiocarp uplifted, pileus lacerated becoming flabelliform or spathulate, imbricate; abhymenial surface brown to yellowish brown, somewhat radially rugulose or wrinkled, obscurely zonate; stipe short and central to somewhat lateral. Generative hyphae are commonly clamped and rarely simple-septate, occasionally covered by dense crystals. Basidiospores globose to subglobose, tuberculate.

Type. CHINA. Yunnan Province, Yimen County, 24°27′N−24°57′N, 101°54′E−102°18′E, elev. 1,036–2,680 m, growing in coniferous and broad-leaved mixed forests, 21.VII.2022, Yuan 16794 (holotype IFP 19545).

Etymology. *Pseudoganbajun* refers to the species being highly morphologically similar to *Th. ganbajun*.

Basidiocarp: Upright, usually solitary, small to medium-sized, up to 10 cm high and 8 cm wide, coriaceous when fresh, tastes mild, slight fragrance when dried. Pileus more or less deeply lacerated becoming flabelliform or spathulate, often imbricate, sometimes proliferating from the center and becoming imbricate and forming a rosette shape, margin flush and thin (0.5–2 mm), and imperceptibly wavy. Abhymenial surface somewhat radially rugulose or wrinkled, obscurely zonate, and brown (6E5–6E6) near the center and becoming yellowish brown (5D4–5D8) near margin; hymenial surface radially rugulose or longitudinally wrinkled, light brown (6D4–6D8) to brown (6E5–6E6) at base and white (6A1) at the margin, inconspicuously tuberculose. Stipe short and central to somewhat lateral, brown (6E5–6E6) to yellowish brown (5D4–5D8), glabrous, cylindrical to flatted or broadened.

Hyphal structure: Hyphal system monomitic; generative hyphae commonly clamped and rarely simple-septate, CB+ in thick-walled hyphae, IKI–; tissues turned brown-black in 3% KOH.

Context: generative hyphae hyaline, thick-walled (< 1 μm), moderately branched, sometimes flexuous and collapsed, subparallel to loosely interwoven, 4–6 μm diam.

Subhymenium: generative hyphae hyaline, slightly thick-walled (< 1 μm), frequently branched often near the clamp connections, occasionally isotypical clamp connections symmetrically growing on both sides of the hyphae, hyphal cells partly short to slightly inflated, occasionally covered by dense crystals, subparallel to loosely interwoven, up to 4–8 μm diam.

Cystidia and cystidioles: Absent.

Basidia: clavate, thin- to slightly thick-walled (< 1 μm), 45–65 × 6–10 μm, clamped at the base, multi-guttulate content sometimes very dense, with four sterigmata, sterigmata 2–6 μm long and 1–2 μm in diam at the base.

Basidiospores: slightly thick-walled (< 1 μm), (5.1–)5.5–7(−7.2) × (4–)4.3–5.5(−6.1) μm (ornamentation excluded), L = 6.47 μm, W = 5.24 μm, Q = 1.22–1.26 (*n* = 60/2), subglobose to irregularly lobed, tuberculate, bluish green in 3% KOH and in distilled water, CB+, IKI–, tuberculi usually isolated, sometimes in groups of two or more.

Additional specimens (paratypes) examined: CHINA. Yunnan Province, Eshan County, 24°10′N, 102°45′E, elev. 1,412 m, growing on the ground, 21.VII.2022, Yuan 16780 (IFP 19546); Muding County, 25°18′N, 101°32′E, elev. 1140–2897 m, growing on the ground, 21.VII.2022, Yuan 16835 (IFP 19547); Shiping County, growing on the ground, 23°42′N, 102°29′E, 1,420–2,551.3 m, 19.VII.2022, Yuan 16771 (IFP 19548); Xundian County, Hekou, 24°27′N−24°57′N, 101°54′E−102°18′E, elev. 76.4–2,354 m, growing in angiosperm and *Pinus* spp. mixed forest, 19.VII.2022, Yuan 16733 (IFP 19549).

Notes: Six samples (Yuan 16780, 16733, 16835, 16771, and 16794) together with two sequences downloaded from GenBank, which were labeled “*Thelephora* cf. *ganbajun*” from Xiangyun County and Yunlin County, Yunnan Province, formed a clade in the phylogenetic tree (Figure 1). In morphology, the flabelliform or spathulate and imbricate pilei of similar size as well as brown to yellowish brown stipe make *Th. pseudoganbajun* easily confused with *Th. ganbajun*. However, a brown to yellowish brown abhymenial surface, a light brown to brown hymenial surface, clamps symmetrically growing on subhymenial hyphae, and the absence of cystidia make the former different from *Th. ganbajun* (Zang, 1987). The phylogenetic tree also shows a close relationship between *Th. pseudoganbajun* and *Th. vialis*. They share similar characteristics in having tuberculate ornamentation of basidiospores and flabelliform or spathulate pilei. However, *Th. vialis* differs from *Th. pseudoganbajun* by a pallid yellowish to pale dull brown abhymenial surface and a pallid yellowish to grayish brown hymenial surface, as well as no clamps symmetrically growing on the subhymenial hyphae (Corner, 1968). Morphologically, flabelliform or spathulate and imbricate pilei and solitary to concrescent basidiocarps, make *Th. pseudoganbajun* similar to *Th. anthocephala*. However, *Th. anthocephala* differentiates from *Th. pseudoganbajun* by a ferruginous or purplish abhymenial surface and a dark brown to the grayish violet hymenial surface, a subtomentose stipe, as well as purplish umber and bigger echinulate basidiospores (7–11 × 5–8.5 μm in *Th. anthocephala* vs. 5.1–7.2 × 4–6.1 μm in *Th. pseudoganbajun* Corner, 1968).

**Figure 14:**
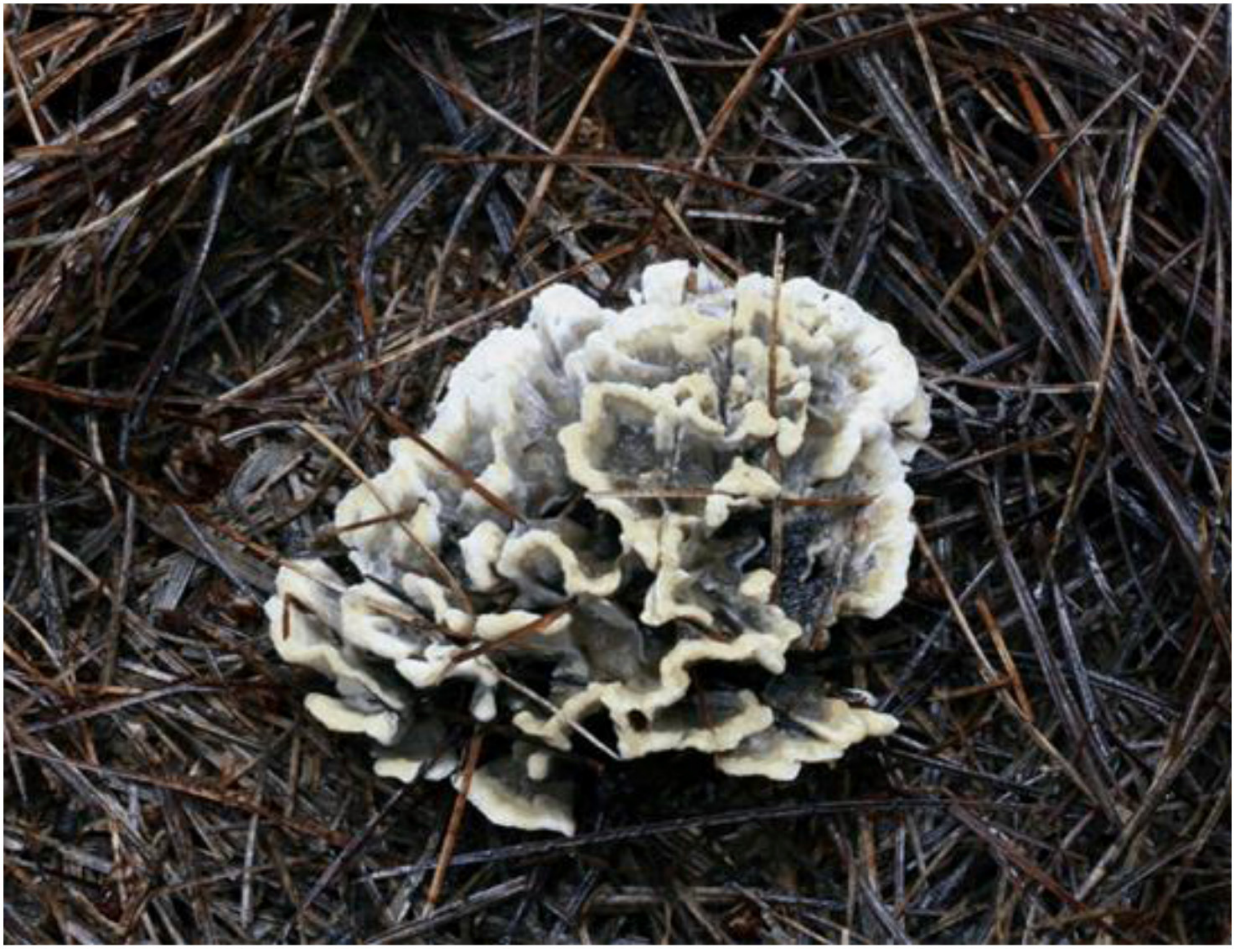
Basidiocarp of *Thelephora ganbajun* (IFP 19554).

**Figure 15:**
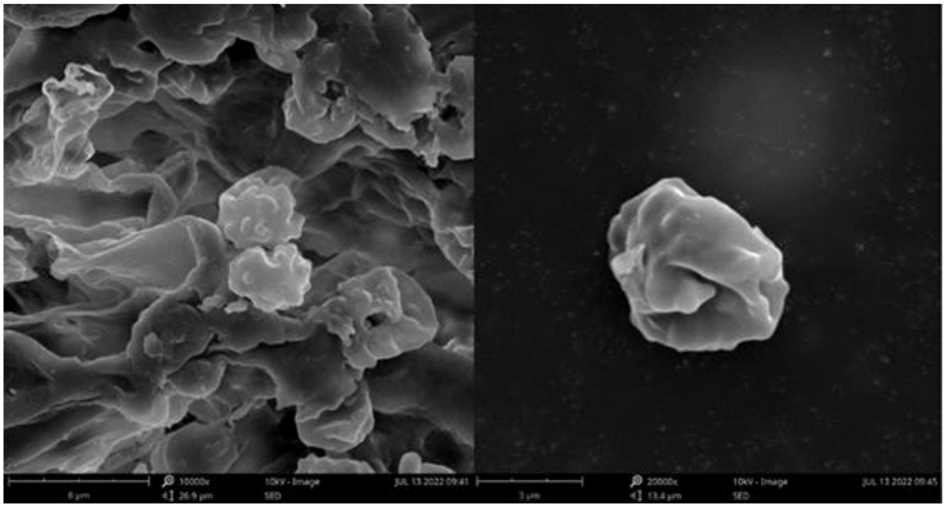
SEM of basidiospores of *Thelephora ganbajun* (IFP 19554).

**Figure 16:**
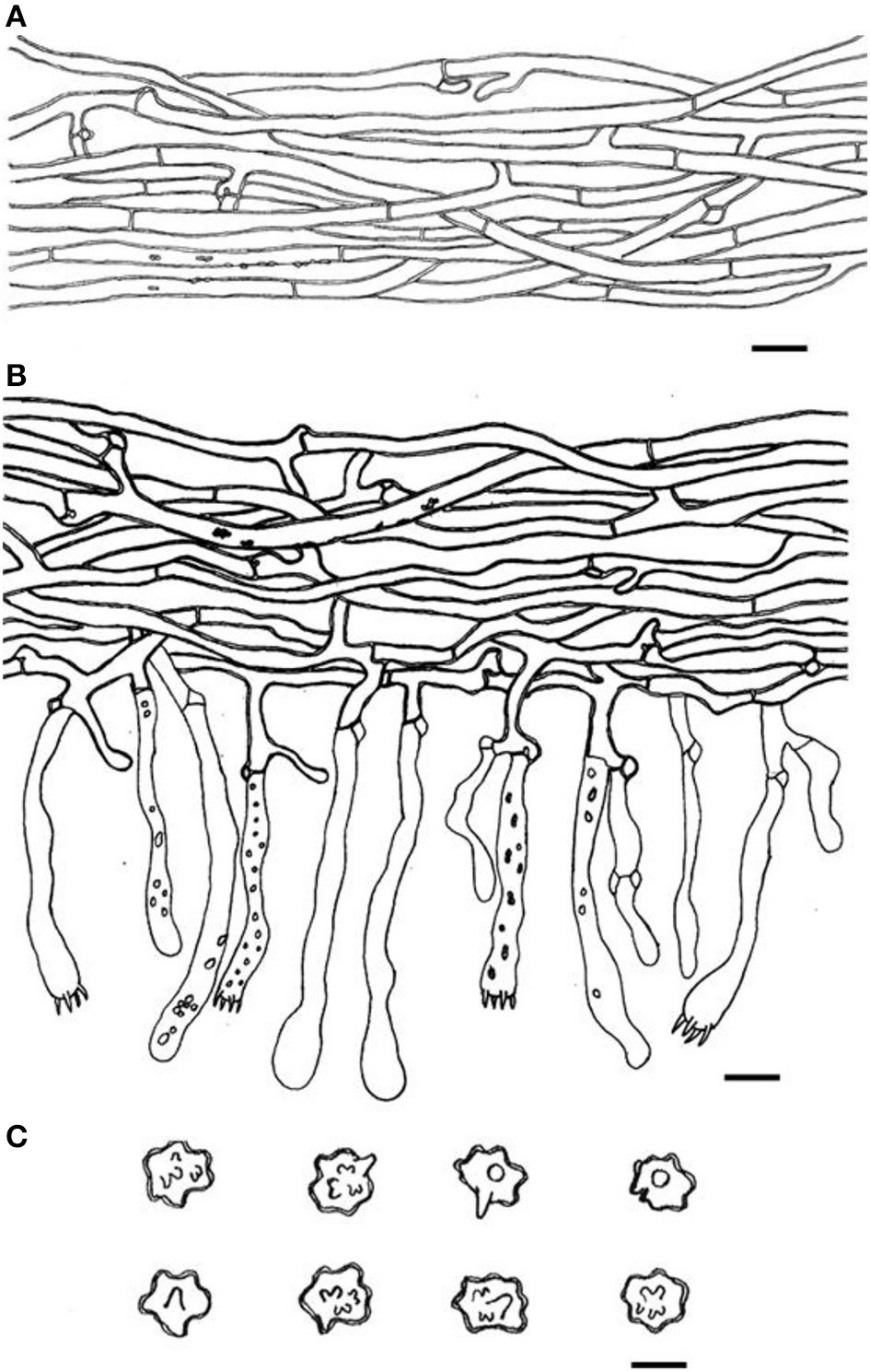
Microscopic structures of *Thelephora ganbajun* (IFP 19554). **(A)** Hyphae from pileal context; **(B)** section of hymenium and subhymenium; **(C)** basidiospores. Scale bars: **(A, B)** = 10 μm; **(C)** = 5 μm.

***Thelephora ganbajun*** M. Zang, Acta Bot. Yunn. 9(1): 85 (1987) (Figures 14–16).

Basidiocarp: Upright, usually solitary, small to medium-sized, up to 14 cm high and 15 cm wide, coriaceous when fresh, tastes mild, coriaceous, yeast powder flavor when dried. Pileus more or less deeply lacerate becoming flabelliform, often imbricate, sometimes proliferating from the center and becoming imbricate and forming a rosette shape, and margin flush and thin (0.5–2 mm). Abhymenial surface somewhat smooth, distinctly zonate, gray (GLEY1 4/N−6/N) to dark brown (10YR 2/1–2/2) near the center and becoming grayish yellow (3C3–3C5) to white (3A1) gradually toward margin; hymenial surface gray (GLEY1 4/N−6/N) to black (GLEY1 2.5/N) near the base and oyster white (2C2) at the margin, inconspicuously gray (GLEY1 4/N−6/N) to black (GLEY1 2.5/N) tuberculose at the base. Stipe short, central to somewhat lateral, multi-branched, brown (6E5–6E6) to yellowish brown (5D4–5D8), glabrous, cylindrical to flatted or broadened.

Hyphal structure: Hyphal system monomitic; generative hyphae commonly clamped and rarely simple-septate, CB+ in thick-walled hyphae, IKI–; tissues turned brown-black in 3% KOH.

Context: Generative hyphae hyaline, thick-walled (< 1 μm), moderately branched, sometimes flexuous and collapsed, subparallel to loosely interwoven, 3–5 μm in diam.

Subhymenium: Generative hyphae hyaline, slightly thick-walled (< 1 μm), frequently branched often near the clamp connections, slightly inflated, occasionally covered by dense crystals, loosely interwoven, up to 3–6 μm diam.

Cystidia: Clavate, 50–85 × 6–8 μm, the length is 1–1.5 times that of basidia, clamped at base; cystidioles absent.

Basidia: Utriform to subcylindrical, thin- to slightly thick-walled (< 1 μm), 25–55 × 6–8 μm, clamped at the base, multi-guttulate content sometimes very dense, occasionally covered by dense crystals, with four sterigmata, sterigmata 4–7 μm long and 1–2 μm in diam at the base.

Basidiospores: slightly thick-walled (< 1 μm), (5–)5.5–7(−7.5) × (4.5–)5–6(−6.7) μm (ornamentation excluded), L = 6.61 μm, W = 5.70 μm, Q = 1.16–1.20 (*n* = 60/2), subglobose to irregularly lobed, often containing a single guttule, tuberculate, bluish green in 3% KOH and in distilled water, CB+, IKI–, tuberculi usually isolated, sometimes in groups of two or more.

Specimens examined: CHINA. Yunnan Province, Jinning District, 24°40′N, 102°35′E, elev. 2,200–2,648 m, 20.VII.2022, Yuan 16765 (IFP 19550); Lufeng City, 25°09′N, 104°04′E, elev. 1,300–2,754 m, 21.VII.2022, Yuan 16817 (IFP 19551); Maming township, 25°25′N, 103°57′E, elev. 2,034 m, growing in coniferous forests, 18.VII.2022, Yuan 16715 (IFP 19552); Shiping County, 23°42′N, 102°29′E, elev. 1,420–2,551.3 m. 20.VII.2022, Yuan 16769 (IFP 19553); Xiangyun County, 41°68′N, 123°47′E, elev. 2,000 m, growing in coniferous forests, 19.IX.2019, Yuan 14373 & 14374 (IFP 19554 & 19555); Xundian County, 25°33′N, 103°15′E, elev. 1,450–3,294 m, 19.VII.2022, Yuan 16749 (IFP 19556); Yiliang County, 24°55′N, 103°08′E, elev. 1,500–1,800 m, 19.VII.2022, Yuan 16756 (IFP 19557).

Notes: *Thelephora ganbajun* was initially described in Yunnan, China (Zang, 1987). Wang et al. (2017b) sequenced the ITS gene of the type specimen preserved in the Cryptogamic Herbarium of the Kunming Institute of Botany, Chinese Academy of Sciences (HKAS-KUN), and uploaded it to the NCBI database. According to the original description, *Th. ganbajun* possesses echinulate basidiospores (7–12 × 6–8 μm); Li et al. (2020) re-examined the type specimen of *Th. ganbajun* and revealed that *Th. ganbajun* possesses much smaller tuberculate basidiospores (4.5–6 × 4.2–5 μm) compared to the original description. In this study, we collected eight samples from different regions of Yunnan Province and identified them as *Th. ganbajun* based on phylogenetic and morphological evidence. In an effort to understand the morphological characters of *Th. ganbajun*, the verification of our eight samples also reveals the smaller sizes of basidiospores (5–7.5 × 4.5–6.7 μm), as well as tuberculate ornamentation. Therefore, we have added some morphological features of *Th. ganbajun* and provided illustrations.

## Key to the new and allied species from China

1. Cystidia present………………………………2
1. Cystidia absent………………………………3
2. Cystidia tubular or septate……………*Th. grandinioides*
2. Cystidia clavate………………………*Th. ganbajun*
3. Isotypical clamps occasionally present symmetrically growing on both sides of the subhymenial hyphae...………………………*Th. pseudoganbajun*
3. Clamps not symmetrically grow on subhymenial hyphae…..4
4. Basidiocarp like wavy cloud………………..*Th. nebula*
4. Basidiocarp not like wavy cloud…………………....5
5. Hymenial surface dark-colored to almost black…..*Th. aquila*
5. Hymenial surface more or less lilac to violet-gray………6
6. Abhymenial surface grayish to black……*Th. austrosinensis*
6. Abhymenial surface gray to greenish gray...……………………………*Th. glaucoflora*

## Discussion

The three-gene (ITS + nLSU + mtSSU) phylogenetic analysis provided an improved resolution at the interspecific level. The tree showed that the phylogenetic clades obtained higher support at the species level, but relatively low support in the deeper nodes, which is consistent with previous results (Ramírez-López et al., 2013, 2015; Khalid and Hanif, 2017; Das et al., 2018; Li et al., 2020).

The phylogenetic tree revealed the relationships among *Thelephora ganbajun, Th. austrosinensis, Th. grandinioides, Th. Vialis*, and the four new species. These eight species clustered in a clade and obtained full support, indicating that they have a close phylogenetic relationship. They share some common morphological characteristics, including flabelliform to imbricate pilei proliferating from a common base, zonate abhymenial surface, generative hyphae more or less covered by crystals, and relatively small, tuberculate basidiospores (5–8 × 4–7 μm), but are significantly distinguished in terms of molecular sequences and morphological characteristics. We provide a key to the new and allied species from China. *Th. vialis* is not included in the key, because our preliminary study shows that the name “*Th. vialis*” may represent another species in China.

The specimens involved in this study were mainly collected from subtropical forests, where the elevation is relatively high (800–2,200 m) and the aphyllophoroid fungi are very rich (He et al., 2011; Wu et al., 2016, 2020, 2022; Cui et al., 2018, 2019; Deng C. Y. et al., 2020; Deng W. Y. et al., 2020; Dai et al., 2021; Ma et al., 2022). The forests are primarily dominated by broad-leaved trees such as Fagaceae, *Castanopis* spp., and a small portion of Pinaceae trees. As ectomycorrhizal fungi, these species may be associated with tree species of Fagaceae and/or Pinaceae.

Up to now, more than 600 ITS sequences named “*Thelephora ganbajun*” have been submitted to NCBI and UNITE databases. The previous study has shown that some selected sequences formed five distinct clades in the ITS phylogenetic tree (Li et al., 2020), and the reference sequence from the type specimen (HKAS 14735) nested in the clade 1, which represents the true *Th. ganbajun*. In this study, some sequences named “*Th. ganbajun*” from GenBank, for instance, KY245247, KY245255, EU696860, and EU696931, have sequence similarities with the type Yuan 16794 ranging from 99.8 to 100% and were identified as *Th. pseudoganbajun*. Some sequences, for example, EU696831, EU696818, EU696871, and EU696881, have sequence similarities with the type Dai 13623A ranging from 99.69 to 99.08% and were identified as *Th. glaucoflora*. However, the other two new species, *Th. aquila* and *Th. nebula*, were not identified with those known sequences from GenBank. More continuous investigations are needed to understand the species diversity of this group of fungi.

## Data availability statement

The datasets presented in this study can be found in online repositories. The names of the repository/repositories and accession number(s) can be found in the article/supplementary material.

## Author contributions

H-SY conceived the study and gave the final approval of the manuscript to be published. H-SY and Y-LW performed the investigation and sample collection. S-RY conducted the experiments, analyzed the data, and wrote the original draft. All authors revised, read, and approved the final manuscript.
